# Significance of Metabolic Tumor Volume at Baseline and Reduction of Mean Standardized Uptake Value in ^18^F-FDG-PET/CT Imaging for Predicting Pathological Complete Response in Breast Cancers Treated with Preoperative Chemotherapy

**DOI:** 10.1245/s10434-019-07325-8

**Published:** 2019-04-02

**Authors:** Tomoko Higuchi, Yukie Fujimoto, Hiromi Ozawa, Ayako Bun, Reiko Fukui, Yoshimasa Miyagawa, Michiko Imamura, Kazuhiro Kitajima, Koichiro Yamakado, Yasuo Miyoshi

**Affiliations:** 1 Department of Surgery, Division of Breast and Endocrine Surgery, Hyogo College of Medicine, Nishinomiya, Hyogo Japan; 2 Department of Nuclear Medicine and PET Center, Hyogo College of Medicine, Nishinomiya, Hyogo Japan

## Abstract

**Background:**

The usefulness of ^18^F-fluorodeoxyglucose-positron emission tomography/computed tomography for evaluating the treatment efficacy of breast cancers is well-established; however, the predictive values of parameters such as metabolic tumor volume (MTV) and total lesion glycolysis (TLG) remain unknown.

**Methods:**

This study examined 199 breast cancers treated with primary systemic chemotherapy (PSC) followed by operation, and determined the values of maximum standardized uptake value (SUV_max_), peak SUV (SUV_peak_), mean SUV (SUV_mean_), MTV, and TLG at baseline. Among these cases, data on early changes in these metabolic parameters in 70 breast cancers were also assessed.

**Results:**

A pathological complete response (pCR) was achieved in 64 breast cancers. Breast cancers with low MTV at baseline had a significantly higher pCR rate than breast cancers with high MTV (47.9% vs. 23.4%; *p* *=* 0.0005). High reduction rates (∆) of SUV_max_ (*p* *=* 0.0001), SUV_peak_ (*p* *=* 0.0001), and SUV_mean_ (*p* *<* 0.0001) resulted in an increased pCR compared with those for low ∆. The pCR rate was highest for the combination of low MTV and high ∆SUV_mean_ (86.7%), and lowest for high MTV and low ∆SUV_mean_ (15.4%); the remaining combinations were intermediate (58.6%; *p* *<* 0.0001). The combination of low MTV at baseline and high ∆SUV_mean_ was a significant and independent predictor for pCR (odds ratio 28.63; 95% confidence interval 1.94–422.42; *p* *=* 0.0146) in multivariable analysis.

**Conclusions:**

Low levels of MTV at baseline and a high reduction of SUV_mean_ after PSC was significantly associated with pCR. These findings suggest the usefulness of these metabolic parameters for predicting the treatment efficacy of breast cancers.

**Electronic supplementary material:**

The online version of this article (10.1245/s10434-019-07325-8) contains supplementary material, which is available to authorized users.

Pathological complete response (pCR), defined as the lack of residual cancer after neoadjuvant chemotherapy (NAC), has been established as a surrogate marker for excellent prognosis of operable breast cancers. Small tumor size, higher tumor grade, and high proliferative activity are significant predictors for a high rate of pCR.^1^ However, the sensitivity and specificity of predicting pCR using these clinical factors are not high^2^^,^^3^ and the identification of more precise predictive factors is a critical issue.

In addition to the diagnostic ability of ^18^F-fluorodeoxyglucose-positron emission tomography/computed tomography (FDG-PET/CT),^4^ the usefulness of the maximum standardized uptake value (SUV_max_) on FDG-PET for predicting the prognosis of operable breast cancers is well established.^5^^–^9 The value of SUV_max_ as a predictive tool for treatment efficacy was reported in metastatic^10^ and early breast cancers.^11^ In the NAC setting, a significant association between an increased SUV_max_ and a high rate of pCR has been reported.^12^^,^^13^ In addition to baseline values, changes in these values after treatment are significantly associated with the response to chemotherapy.^14^ Since altered glucose metabolism occurs earlier than tumor shrinkage,^15^ it may be possible to evaluate the treatment efficacy as early as after one or two treatment cycles.

SUV_max_ levels have been reported to be inaccurate compared with the actual uptake of FDG, due to the partial volume effect.^16^ Since volume-based parameters on FDG-PET, including metabolic tumor volume (MTV) and total lesion glycolysis (TLG), evaluate not only metabolic activity but also total tumor burden, recent studies have focused on these metabolic parameters rather than on SUV_max_ for predicting the response to chemotherapy. Although early reductions of SUV_max_ are reportedly associated with improved response to NAC,^17^^,^^18^ which metabolic parameter offers the most precise prediction remains unknown. In addition, the superiority of baseline or early response to treatment PET data is also undetermined.

The present study explored the predictive values of SUV_max_, SUV_peak_, SUV_mean_, and volume-based parameters, including MTV and TLG, at baseline in breast cancers treated with primary systemic chemotherapy (PSC). Additionally, early changes in these parameters after treatment were also investigated in terms of their relationships with treatment efficacy.

## Patients and Methods

### Patient Recruitment

This retrospective study constitutively recruited a total of 267 breast cancer patients who underwent surgery after preoperative chemotherapy between October 2008 and May 2018. Among these participants, 194 patients who underwent FDG-PET/CT before starting PSC were selected. Since five patients had bilateral breast cancers, we analyzed a total of 199 breast cancers in 194 patients. We evaluated response in the primary sites (breast), and nine patients with stage IV who had PSC followed by operation were also included. We also obtained FDG-PET/CT data after the start of PSC for 69 patients (70 breast cancers).

The Ethics Committee of Hyogo College of Medicine approved the present study (numbers 1818 and 1708), and written informed consent was obtained from all 69 participants who underwent FDG-PET/CT after the start of PSC (number 1708, UMIN000030408). In the remaining patients, only baseline FDG-PET/CT data from clinical practice were used and offered no risk to participants; thus, written informed consent was not required (number 1818).

### Chemotherapy Regimen and Evaluation of Pathological Response

Preoperative chemotherapies involving anthracycline-containing, taxane-based, sequential use of anthracycline-containing and taxane, and unspecified regimens were administered in 6, 48, 137, and 3 patients, respectively. Concurrent use of trastuzumab with chemotherapy was administered to 64 patients. Pathological examinations of the whole area of pre-existing breast cancer lesions were performed, and pCR was defined as complete absence of invasive cancer cells in the breast.^19^

### ^18^F-Fluorodeoxyglucose-Positron Emission Tomography/Computed Tomography (FDG-PET/CT) Procedure

FDG-PET/CT was performed using a Gemini GXL16 or Gemini TF64 PET/CT scanner (Philips Medical Systems, Eindhoven, The Netherlands) following injection of 4.0 or 3.0 MBq/kg body weight FDG for the GXL16 and TF64, respectively. Scanning images were obtained approximately 60 min after injection, as described previously.^20^ The 194 patients underwent FDG-PET/CT examination before starting PSC, of whom 69 (70 breast cancers) underwent a repeat FDG-PET/CT examination after starting chemotherapy. We obtained FDG-PET/CT data after one cycle of PSC (2–3 weeks after the start of chemotherapy), except for one patient whose data were obtained after two cycles (electronic supplementary data).

### Imaging Analyses

To quantify ^18^F-FDG uptake, the SUVs were measured. We set the volume of interest (VOI) as the area in which FDG accumulated in the breast, along the margin of tumor uptake. The SUV was calculated as the regional radioactivity concentration (Bq/mL)/[injected dose (Bq)/patient weight (g)] in the most intense area of ^18^F-FDG accumulation (a region of interest [ROI]). We selected the region containing the tumor in which the FDG in the breast was accumulated, as observed on the image, and set a target VOI manually in the breast cancer primary lesion with FDG accumulation. The maximum value of SUV in the VOI was defined as the SUV_max_, and the volume of voxels of ≥ 40% of the SUV_max_ in the VOI was defined as the MTV.^21^^–^23 The SUV_peak_ was defined as the average activity concentration within a 1 cm^3^ spherical VOI centered on the ‘hottest focus’ within the primary tumor. The average SUV value in the voxel that showed ≥ 40% was defined as the SUV_mean_ and TLG was defined as MTV × SUV_mean_. These parameters were all automatically calculated by the computer software package GI-PET (AZE Co., Ltd, Tokyo, Japan). Harmonization of data in different PET/CT systems was performed using phantom data.

The percentage changes (∆%) of PET data at baseline and after the start of PSC in each of the five parameters were calculated as follows: percentage change (∆%) = (delayed parameter − baseline parameter)/baseline parameter × 100.

### Statistical Analysis

The associations of clinicopathological characteristics between breast cancers that achieved pCR and those that did not were analyzed using the Fisher’s exact or Wilcoxon rank-sum tests. The relationships between pCR and levels of each metabolic parameter were calculated using Fisher’s exact tests, and logistic regression was used to obtain odds ratios (ORs) and 95% confidence intervals (CIs) by univariable and multivariable analyses of clinical factors or metabolic parameters and pCR. Statistical significance was set at *p* *<* 0.05. All statistical calculations were performed using JMP Pro 13 (SAS Institute Inc., Cary, NC, USA).

## Results

### Relationships Between Clinicopathological Factors and Pathological Response

We defined pCR and non-pCR in 64 and 135 breast cancers, respectively. The pCR rates were significantly higher in those with small tumor size, higher nuclear grade, estrogen receptor (ER)-negative/human epidermal growth factor receptor 2 (HER2)-negative (triple-negative [TN]) and HER2-positive subtypes, high levels of Ki67, and an anthracycline and taxane regimen (electronic supplementary Table 1 and data). The SUV_max_, SUV_peak_, and SUV_mean_ were significantly associated with nuclear grade and Ki67 expression levels. There were significant associations between tumor size and all parameters except SUV_max_, and the subtypes were significantly associated with all parameters except TLG. Lymph node metastasis was significantly associated with MTV and TLG (electronic supplementary Table 2).

### Determination of the Optimal Cut-Off Values for Pathological Complete Response (pCR) of Metabolic Parameters by PET/CT at Baseline and During Primary Systemic Chemotherapy

**Fig. 1:**
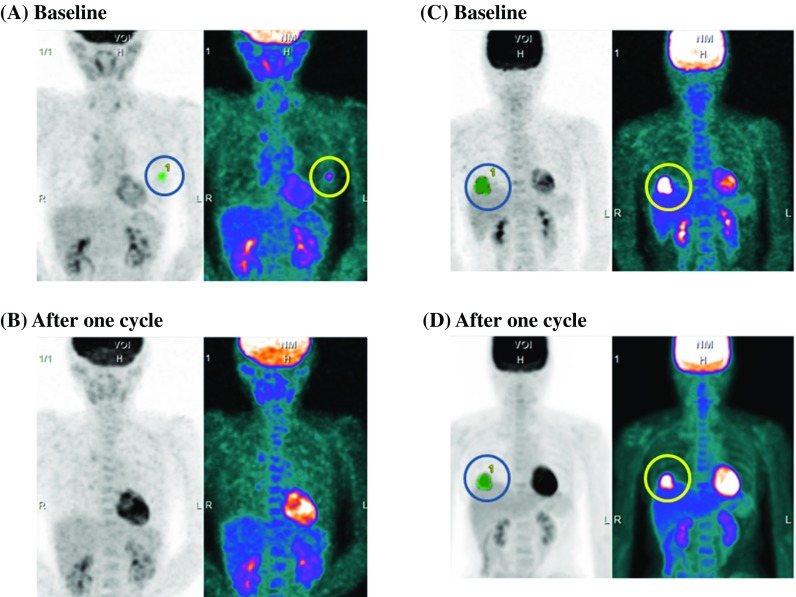
Representative cases of FDG accumulation in breast cancers at baseline and after the start of primary systemic chemotherapy. **a** Baseline and **b** after one treatment cycle in a case of breast cancer that achieved a pCR. **c** Baseline and **d** after one treatment cycle of a case of breast cancer that did not achieve a pCR. *FDG* fluorodeoxyglucose, *pCR* pathological complete response

Representative cases of PET imaging are shown in Fig. 1. The FDG uptake detected in the left breast at baseline was diminished after one cycle of chemotherapy (Fig. 1a, b) in patients who achieved pCR; however, the uptake of FDG at baseline in the right breast remained after one cycle of chemotherapy (Fig. 1c, d). The cut-off values of SUV_max_, SUV_peak_, SUV_mean_, MTV, and TLG at baseline for pCR were determined using receiver operating characteristic (ROC) curves calculated using the Youden index for the areas under the curve (AUC) (electronic supplementary Fig. 1). Similar methods were used to determine the cut-off values of the reduction rate in each metabolic parameter for pCR (electronic supplementary Fig. 2).

### Associations Between pCR After PSC and Each Metabolic Parameter

Breast cancers with high baseline levels of SUV_max_, SUV_peak_, and SUV_mean_ had a significantly higher rate of pCR than breast cancers with low levels. However, the frequency of pCR was significantly higher for low baseline levels of MTV and TLG (electronic supplementary Fig. 3). Of these baseline parameters, MTV was the most significant predictor for pCR (47.9% vs. 23.4%; *p* *=* 0.0005).

Similarly, breast cancers with high reduction rates had significantly higher frequencies of pCR for ∆SUV_max_, ∆SUV_peak_, ∆SUV_mean_, and ∆TLG, but not ∆MTV, in which the significance was marginal (electronic supplementary Fig. 4). The difference in pCR rates between the two groups was most significant for the ∆SUV_mean_ (72.2% vs. 23.5%; *p* < 0.0001).

### Univariable and Multivariable Analyses of pCR, Including Metabolic Parameters at Baseline and During Treatment

First, we analyzed data of all 199 breast cancers, including metabolic parameters at baseline. Tumor size, nuclear grade, Ki67 expression levels, subtypes, chemotherapy regimen, and all metabolic parameters at baseline, including SUV_max_, SUV_peak_, SUV_mean_, MTV, and TLG, were significantly associated with pCR in univariable analysis (Table 1). The multivariable analyses included these clinical factors with one of the metabolic parameters. As shown in Table 1, baseline MTV was significantly associated with pCR in multivariable analysis (OR 0.30, 95% CI 0.11–0.84; *p* *=* 0.0212).

**Table 1:** Univariable and multivariable analyses of the clinicopathological characteristics and metabolic parameters at baseline for pathological complete response in 199 breast cancers

	*n*	Univariable	*p* value	Multivariable	*p* value
		[OR (95% CI)]		[OR (95% CI)]	
Menopausal status					
Premenopausal	80	1.00			
Postmenopausal	119	1.59 (0.85–2.96)	0.1447		
T size (cm)					
≤ 2.0	43	1.00		1.00	
> 2	156	0.40 (0.20–0.80)	0.0093	0.40 (0.13–1.20)	0.1024
Lymph node metastasis					
Negative	104	1.00			
Positive	95	0.65 (0.36–1.20)	0.1677		
Nuclear grade					
1	74	1.00		1.00	
2 + 3	115	5.76 (2.62–12.66)	< 0.0001	3.14 (1.09–9.04)	0.0342
Ki67 expression levels^a^					
Low	50	1.00		1.00	
High	139	9.74 (2.89–32.89)	0.0002	20.58 (1.55–237.07)	0.0219
Subtypes^b^					
TN	48	1.00		1.00	
Luminal A	31	0.04 (0.01–0.34)	0.0029	2.23 (0.08–65.78)	0.6418
Luminal B	49	0.18 (0.06–0.50)	0.0010	0.20 (0.06–0.67)	0.0114
Luminal-HER2	36	0.57 (0.23–1.41)	0.2198	1.18 (0.34–4.09)	0.7908
HER2	33	3.43 (1.32–8.91)	0.0114	5.45 (1.53–19.41)	0.0089
Chemotherapy regimen					
Taxane	48	1.00		1.00	
Anthracycline and taxane	142	3.26 (1.42–7.47)	0.0053	2.83 (0.92–8.77)	0.0707
SUV_max_^c^					
Low	60	1.00			
High	139	2.10 (1.04–4.24)	0.0396	Not included	
SUV_peak_^d^					
Low	62	1.00			
High	137	2.24 (1.11–4.51)	0.0249	Not included	
SUV_mean_^e^					
Low	47	1.00			
High	152	2.84 (1.24–6.52)	0.0135	Not included	
MTV^f^					
Low	71	1.00			
High	128	0.33 (0.18–0.62)	0.0005	0.30 (0.11–0.84)	0.0212
TLG^g^					
Low	110	1.00			
High	89	0.43 (0.23–0.81)	0.0092	Not included	

*OR* odds ratio, *CI* confidence interval, *TN* triple-negative, *ER* estrogen-receptor, *HER2* human epidermal growth factor receptor 2, *SUV*_*max*_ maximum standardized uptake value, *SUV*_*peak*_ peak standardized uptake value, *SUV*_*mean*_ mean standardized uptake value, *MTV* metabolic tumor volume, *TLG* total lesion glycolysis

^a^Low < 20%, high ≥ 20%

^b^TN, ER-negative/HER2-negative; Luminal A, ER-positive/HER2-negative with Ki67 < 20%; Luminal B, ER-positive/HER2-negative with Ki67 ≥ 20%; Luminal-HER2, ER-positive/HER2-positive; HER2, ER-negative/HER2-positive

^c^Low < 3.664, high ≥ 3.664

^d^Low < 3.279, high ≥ 3.279

^e^Low < 1.782, high ≥ 1.782

^f^Low < 4.416, high ≥ 4.416

^g^Low < 20.138, high ≥ 20.138

Data of metabolic parameters during treatment in 70 breast cancers were further analyzed. All of ∆SUV_max_, ∆SUV_peak_, ∆SUV_mean_, and ∆TLG were significant predictive factors for pCR in the univariable analysis (Table 2). Since the association between pCR and ∆SUV_mean_ was most significant, we performed multivariable analysis, including only ∆SUV_mean_ as a metabolic parameter, and identified ∆SUV_mean_ as a significant and independent factor, as shown in Table 2 (OR 8.05, 95% CI 1.45–44.80; *p* *=* 0.0173).

**Table 2:** Univariable and multivariable analyses of the clinicopathological characteristics and the reduction of metabolic parameters after the start of chemotherapy for pathological complete response in 70 breast cancers

	*n*	Univariable	*p* value	Multivariable	*p* value
		[OR (95% CI)]		[OR (95% CI)]	
Menopausal status					
Premenopausal	31	1.00			
Postmenopausal	39	1.28 (0.50–3.29)	0.6110		
T size (cm)					
≤ 2.0	14	1.00		1.00	
> 2	56	0.11 (0.02–0.53)	0.0060	0.07 (0.004–1.03)	0.0522
Lymph node metastasis					
Negative	29	1.00			
Positive	41	0.64 (0.24–1.66)	0.3538		
Nuclear grade					
1	16	1.00		1.00	
2 + 3	50	11.42 (2.33–55.88)	0.0026	14.89 (1.07–207.75)	0.0446
Ki67 expression levels^a^					
Low	9	1.00			
High	58	8.57 (1.01–72.98)	0.0493	Not calculated	
Subtypes^b^					
TN	19	1.00		1.00	
Luminal A	7	Not calculated		Not calculated	
Luminal B	18	0.09 (0.02–0.45)	0.0030	0.11 (0.01–0.84)	0.0335
Luminal-HER2	11	0.55 (0.12–2.56)	0.4494	0.34 (0.04–3.01)	0.3352
HER2	14	1.69 (0.34–8.40)	0.5197	0.71 (0.07–6.79)	0.7692
Chemotherapy regimen					
Taxane	6	1.00			
Anthracycline and taxane	63	5.50 (0.61–49.80)	0.1294		
∆SUV_max_^c^					
Low	33	1.00			
High	37	7.39 (2.55–21.39)	0.0002	Not included	
∆SUV_peak_^d^					
Low	33	1.00			
High	37	7.39 (2.55–21.39)	0.0002	Not included	
∆SUV_mean_^e^					
Low	34	1.00			
High	36	8.45 (2.88–24.81)	0.0001	8.05 (1.45–44.80)	0.0173
∆MTV^f^					
Low	26	1.00			
High	44	2.49 (0.91–6.79)	0.0756	Not included	
∆TLG^g^					
Low	32	1.00			
High	38	6.50 (2.27–18.62)	0.0005	Not included	

*OR* odds ratio, *CI* confidence interval, *TN* triple-negative, *ER* estrogen receptor, *HER2* human epidermal growth factor receptor 2, *SUV*_*max*_ maximum standardized uptake value, *SUV*_*peak*_ peak standardized uptake value, *SUV*_*mean*_ mean standardized uptake value, *MTV* metabolic tumor volume, *TLG* total lesion glycolysis, ∆ reduction rate

^a^Low < 20%, high ≥ 20%

^b^TN, ER-negative/HER2-negative; Luminal A, ER-positive/HER2-negative with Ki67 < 20%; Luminal B, ER-positive/HER2-negative with Ki67 ≥ 20%; Luminal-HER2, ER-positive/HER2-positive; HER2, ER-negative/HER2-positive

^c^Low < − 56.3, high ≥ − 56.3

^d^Low < − 55.1, high ≥ − 55.1

^e^Low < − 55.8, high ≥ − 55.8

^f^Low < − 22.2, high ≥ − 22.2

^g^Low < − 65.0, high ≥ − 65.0

### Predictive Ability of pCR for the Combination of Baseline Metabolic Tumor Volume (MTV) and ∆SUV_mean_ Parameters

**Fig. 2:**
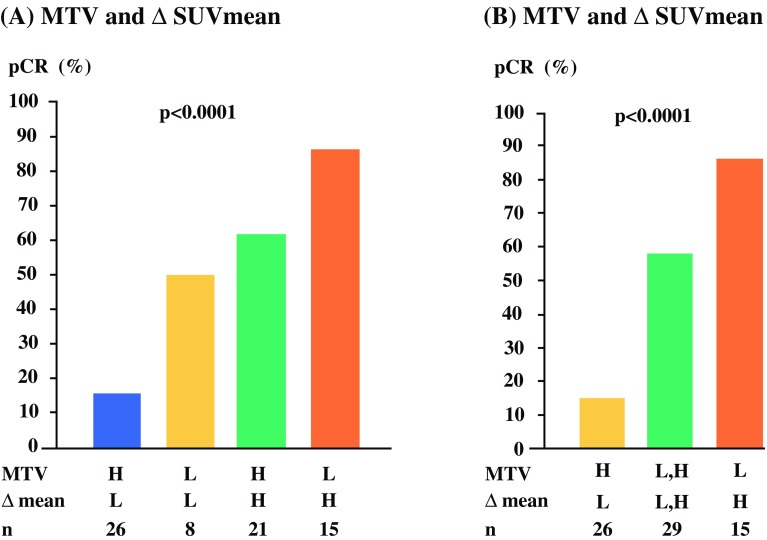
pCR rates according to the combination of MTV at baseline and ∆SUV_mean_. The pCR rates of **a** four groups and **b** three groups after combining both high and both low MTV and ∆SUV_mean_. *pCR* pathological complete response, *MTV* metabolic tumor volume, *SUV*_*mean*_ mean standardized uptake value, *H* high, *L* low, *∆* reduction rate

Since both baseline MTV and ∆SUV_mean_ were significantly associated with pCR, we further analyzed the combination of these parameters. The pCR rate was highest for low baseline MTV and high ∆SUV_mean_ (86.7%), and lowest for high baseline MTV and low ∆SUV_mean_ (15.4%) [Fig. 2a]. Since breast cancers with high baseline MTV and high ∆SUV_mean_, as well as those with low baseline MTV and low ∆SUV_mean_, showed intermediate pCR rates (61.9% and 50%, respectively), we further combined these two intermediate groups in Fig. 2b (pCR rate 58.6%). In multivariable analysis, the combination of baseline MTV and ∆SUV_mean_ was a significant and independent predictor of pCR (OR 28.63, 95% CI 1.94–422.42; *p* *=* 0.0146 for low baseline MTV and high ∆SUV_mean_) (Table 3).

**Table 3:** Univariable and multivariable analyses of the clinicopathological characteristics and the combination of MTV at baseline with the reduction of SUV_mean_ in 70 breast cancers

	*n*	Univariable analysis	*p* value	Multivariable analysis^a^	*p* value
		[OR (95% CI)]		[OR (95% CI)]	
MTV^b^					
Low	47	1.00		1.00	
High	23	0.20 (0.07–0.60)	0.0043	0.18 (0.03–1.19)	0.0749
∆SUV_mean_^c^					
Low	34	1.00			
High	36	8.45 (2.88–24.81)	0.0001	8.05 (1.45–44.80)	0.0173
MTV and ∆SUV_mean_					
High and low	26	1.00		1.00	
Both high, or both low	29	7.79 (2.13–28.49)	0.0019	6.89 (1.10–43.24)	0.0394
Low and high	15	35.75 (5.73–223.00)	0.0001	28.63 (1.94–422.42)	0.0146

*MTV* metabolic tumor volume, *SUV*_*mean*_ mean standardized uptake value, *OR* odds ratio, *CI* confidence interval, *pCR* pathological complete response, ∆ reduction rate

^a^Adjusted for tumor size, nuclear grade, and subtypes that were significantly associated with pCR by univariable analysis in Table 2

^b^Low < 4.416, high ≥ 4.416

^c^Low < − 55.8, high ≥ − 55.8

## Discussion

The results of the present study demonstrated that low levels of baseline MTV and early reduction of SUV_mean_ after the start of treatment were significant and independent predictive factors for a higher rate of pCR in breast cancers treated with PSC. The combination of both parameters predicts pCR more precisely compared with that of baseline MTV or ∆SUV_mean_ alone. The SUV_max_ was a significant predictor of pCR after NAC in 273 breast cancers (OR per one-unit increase 1.09, 95% CI 1.02–1.16; *p* *=* 0.008).^24^ However, consistent with our study, SUV_max_, SUV_peak_, and SUV_mean_ at baseline were not associated with pathological response in previous studies.^21^^,^^25^^–^29 Contrary to SUVs, MTV is a volume-based metabolic parameter that represents both metabolic activity and total tumor burden in each tumor. However, Cho et al. reported no significant association between pCR and baseline TLG or MTV values.^27^ Although Cheng et al. reported no correlation between TLG and pCR,^21^ baseline MTV was marginally associated with pCR in the HER2-negative group (*n* *=* 30; *p* *=* 0.081). The reason for this discrepancy may be the smaller number of participants (*n* *=* 26 and *n* *=* 30) compared with our study (*n* *=* 199).

Although SUV_max_ levels at baseline and after one cycle were not correlated with pCR in 50 TN breast cancers, higher ∆SUV_max_ values were significantly associated with increased pCR in multivariable analysis (OR 7.1; *p* *=* 0.014).^30^ Similarly, early changes in SUV_max_ corrected for lean body mass (SUL_max_) values between those achieving pCR and those not achieving pCR differed significantly in 59 HER2-negative breast cancers (63.0% vs. 32.9%; *p* *=* 0.003).^31^ Further studies demonstrated that not at baseline, but rather ∆SUV_max_ after the start of NAC, was significantly associated with pCR.^25^^,^^28^^,^^29^^,^^32^^,^^33^ In addition, the mean percentage of ∆TLG_30%_ (*p* *=* 0.005), but not ∆MTV_30%_ (*p* *=* 0.262), was significantly greater in the pCR group^27^; however, neither ∆TLG nor ∆MTV were significantly associated with pCR in the report by Cheng et al.^21^ Despite the significant correlation between tumor size reduction rate and the reduction rates of MTV (*p* *=* 0.0004) or TLG (*p* *=* 0.002), but not SUV_max_ (*p* *=* 0.07),^34^ the ∆MTV and ∆TLG might be less useful than ∆SUV_max_ when considering their pCR predicting ability.

Groheux et al. reported the AUC of pCR prediction increased from 0.63 to 0.76 when combined ∆SUV_max_ with genomic grade index (GGI; *p* *=* 0.016) in TN breast cancer patients.^28^ We identified the significance of the combination of baseline MTV and ∆SUV_mean_ in terms of pCR prediction. Interestingly, MTV was significantly associated with tumor size, but not with grade. Conversely, SUV_max_ was significantly associated with grade, but not with tumor size.^33^ Thus, MTV and ∆SUV may be a useful combination for predicting pCR mediating through different mechanisms. Small metabolic tumor size evaluable by MTV and high reduction rate of metabolic activity evaluable by SUV_mean_ may be linked to achieving a pCR. We obtained data regarding metabolic parameters after treatment in 22 breast cancers; all six breast cancers that retained FDG uptake had non-pCR. Even though FDG uptake diminished after treatment, 5 of 16 (31.3%) breast cancers were defined as non-pCR (electronic supplementary data). Thus, the data obtained after treatment may not improve the ability to predict pCR.

We set the optimal cut-off values of ∆SUV_max_, ∆SUV_peak_, and ∆SUV_mean_ at 56.3%, 55.1%, and 55.8%, respectively, and the predictive values of these metabolic parameters were similar. Previous studies reported ∆SUV_max_ cut-off values ranging from 50 to 82.2%.^35^ In addition, we used the average SUV value in the voxel that showed ≥ 40% of SUV_max_ as the SUV_mean_, as used in previous studies.^21^^–^23 In other studies, thresholds of VOI were set to values between 30 and 50%.^27^ Although the best threshold was unknown, we obtained similar results when calculated with other cut-off values and the reproducibility of SUV_mean_ measurement was confirmed by a coauthor (data not shown). Issues regarding which parameter of SUV is most useful, and the best optimal cut-off value or threshold, require confirmation in future studies. In addition, we concluded, based on 70 breast cancers, that the sample size was not enough. Further studies involving large numbers of participants are needed. To our knowledge, this is the first study to demonstrate the useful combination of metabolic parameters obtained by PET for pCR in breast cancers treated with preoperative chemotherapy.

## Conclusions

The combination of baseline MTV and ∆SUV_mean_ precisely predicted the pCR in breast cancers treated with chemotherapy. The predictive value of this combination was independent and strong compared with that of other clinical factors, including tumor size, tumor grade, Ki67 levels, and subtypes. Small metabolic tumor size evaluable by MTV, and a high metabolic activity reduction as determined by SUV_mean_, might be useful for predicting improved pCR in breast cancers treated with preoperative chemotherapy.

## Electronic supplementary material

Below is the link to the electronic supplementary material.

Supplementary material 1 (DOCX 24 kb)

Supplementary material 2 (DOCX 26 kb)

Supplementary material 3 (DOCX 29 kb)

Supplementary material 4 (DOCX 24 kb)

Supplementary material 5 (TIFF 134 kb)

Supplementary material 6 (TIFF 143 kb)

Supplementary material 7 (TIFF 67 kb)

Supplementary material 8 (TIFF 70 kb)
