# Supplementary material for: Significance of Metabolic Tumor Volume at Baseline and Reduction of Mean Standardized Uptake Value in 18F-FDG-PET/CT Imaging for Predicting Pathological Complete Response in Breast Cancers Treated with Preoperative Chemotherapy
Source: Ann Surg Oncol. 2019 Apr 2;26(7):2175–83. doi: 10.1245/s10434-019-07325-8 (PMC6545174; doi:10.1245/s10434-019-07325-8)
Supplement: Supplementary file 1 — Supplementary material 1 (DOCX 24 kb) [file 10434_2019_7325_MOESM1_ESM.docx]

**Supplementary Data**

**Determination of subtypes**

Estrogen receptor (ER) positivity was defined as immunohistochemical staining of ≥ 1% of nuclei of cancer cells. human epidermal growth factor receptor 2 (HER2) status was defined with an immunohistochemical score of 3 or a positive fluorescence in situ hybridization test for those with an immunohistochemical score of 2. Ki67 expression levels were divided into low (< 20%) and high (≥ 20%) by immunohistochemical staining of the nuclei of cancer cells. Breast cancer subtypes were determined as follows: luminal-A, ER-positive and HER2-negative with Ki67-low; luminal-B, ER-positive and HER2-negative with Ki67-high; luminal-HER2, ER-positive and HER2-positive; HER2, ER-negative and HER2-positive; triple negative (TN), ER-negative and HER2-negative.

**FDG PET/CT procedure**

The median duration from the baseline FDG PET/CT to first chemotherapy was 18 (range, 2–111) days. Of these patients, 69 (70 breast cancers) underwent a repeat FDG PET/CT examination after starting chemotherapy. We obtained FDG PET/CT data after one cycle of PSC (two to three weeks after the start of chemotherapy) except for one patient whose data were obtained after two cycles (66 days after the start of chemotherapy). The median duration between the first chemotherapy and the second FDG PET/CT was 20 days (range, 8–66 days).

In 22 patients out of the 69 patients whose data were available during treatment, we further obtained FDG PET/CT data after treatment (before operation). Using 1.5 times the background breast uptake as the threshold, we divided FDG uptake in breast cancers after the final treatment into two groups; i.e., FDG uptake-positive (n=6) and -negative (n=16). All six patients with remaining FDG uptake after treatment achieved non-pCR. On the contrary, 11 (68.8%) out of 16 breast cancers without FDG uptake had pCR.

The median duration from the last chemotherapy to the FDG PET/CT was 16 (range, 3–133) days. The durations from the first FDG PET/CT to operation and from the last chemotherapy to operation were 206.5 (range, 50–764) days and 34 (range, 13–207) days, respectively.
