# Supplementary material for: Significance of Metabolic Tumor Volume at Baseline and Reduction of Mean Standardized Uptake Value in 18F-FDG-PET/CT Imaging for Predicting Pathological Complete Response in Breast Cancers Treated with Preoperative Chemotherapy
Source: Ann Surg Oncol. 2019 Apr 2;26(7):2175–83. doi: 10.1245/s10434-019-07325-8 (PMC6545174; doi:10.1245/s10434-019-07325-8)
Supplement: Supplementary file 2 — Supplementary material 2 (DOCX 26 kb) [file 10434_2019_7325_MOESM2_ESM.docx]

Supplementary Table 1 Clinicopathological characteristics of breast cancers according to the pathological response

Characteristics pCR*^1^ Non-pCR p-value

(n=64) (n=135)

Age (median, range) 54 (30-79) 55 (26-84) 0.602

Menopausal status

Premenopausal 21 (26.3)*^2^ 59 (73.8) 0.165

Postmenopausal 43 (36.1) 76 (63.9)

Tumor size

≤2.0cm 21 (48.8) 22 (51.2) 0.0102

>2cm 43 (27.6) 113 (72.4)

Lymph node metastasis

Negative 38 (36.5) 66 (63.5) 0.175

Positive 26 (27.4) 69 (72.6)

Nuclear grade

1 9 (12.2) 65 (87.8) < 0.0001

2+3 51 (44.3) 64 (55.7)

Unknown 4 (40.0) 6 (60.0)

Ki67 expression levels*^3^

Low 3 (6.1) 46 (93.9) < 0.0001

High 54 (38.8) 85 (61.2)

Not determined 7 (63.6) 4 (36.4)

Subtypes*^4^

TN 21 (43.8) 27 (56.3) < 0.0001

Luminal A 1 (3.2) 30 (96.8)

Luminal B 6 (12.2) 43 (87.8)

Luminal-HER2 11 (30.6) 25 (69.4)

HER2 24 (72.7) 9 (27.3)

Unknown 1 (50.0) 1 (50.0)

Chemotherapy regimen

Taxane 8 (16.7) 40 (83.3) 0.0009

Anthracycline and Taxane 56 (39.4) 86 (60.6)

Others 0 (0) 9 (100)

*^1^pathological complete response; *^2^ (%); *^3^ low < 20%, high ≥ 20%; *^4^ TN, estrogen receptor (ER)-negative/human epidermal growth factor receptor 2 (HER2)-negative; Luminal A, ER-positive/HER2-negative with Ki67< 20%; Luminal B, ER-positive/HER2-negative with Ki67 ≥ 20%, Luminal-HER2, ER-positive/HER2-positive; HER2, ER-negative/HER2-positive.
