# Supplementary material for: Significance of Metabolic Tumor Volume at Baseline and Reduction of Mean Standardized Uptake Value in 18F-FDG-PET/CT Imaging for Predicting Pathological Complete Response in Breast Cancers Treated with Preoperative Chemotherapy
Source: Ann Surg Oncol. 2019 Apr 2;26(7):2175–83. doi: 10.1245/s10434-019-07325-8 (PMC6545174; doi:10.1245/s10434-019-07325-8)
Supplement: Supplementary file 3 — Supplementary material 3 (DOCX 29 kb) [file 10434_2019_7325_MOESM3_ESM.docx]

Supplementary Table 2 Association between clinicopathological characteristics and metabolic parameters at baseline

SUVmax*^1^ SUVpeak*^2^ SUVmean*^3^ MTV*^4^  TLG*^5^

high low p-value high low p-value high low p-value high low p-value high low p-value

Menopausal status

Premenopausal 58(72.5) 22(27.5) 0.5323 57(71.3) 23(28.7) 0.6400 63(78.8) 17(21.2) 0.6104 51(63.8) 29(36.2) 1.0000 35(43.8) 45(56.2) 0.8847

Postmenopausal 81(68.1) 38(31.9) 80(67.2) 39(32.8) 89(74.8) 30(25.2) 77(64.7) 42(35.3) 54(45.4) 65(54.6)

T size

≤2.0cm 25(58.1) 18(41.9) 0.0636 23(53.5) 20(46.5) 0.0166 27(62.8) 16(37.2) 0.0251 11(25.6) 32(74.4) <0.0001 6(13.9) 37(86.1) <0.0001

>2cm 114(73.1) 42(26.9) 114(73.1) 42(26.9) 125(80.1) 31(19.9) 117(75.0) 39(25.0) 83(53.2) 73(46.8)

Lymph node metastasis

Negative 70(67.3) 34(32.7) 0.4425 70(67.3) 34(32.7) 0.6485 77(74.0) 27(26.0) 0.5043 59(56.7) 45(43.3) 0.0260 39(37.5) 65(62.5) 0.0337

Positive 69(72.6) 26(27.4) 67(70.5) 28(29.5) 75(78.9) 20(21.1) 69(72.6) 26(27.4) 50(52.6) 45(47.4)

Nuclear grade

1 42(56.8) 32(43.2) 0.0003 40(54.1) 34(45.9) <0.0001 50(67.6) 24(32.4) 0.0115 50(67.6) 24(32.4) 0.3549 33(44.6) 41(55.4) 1.0000

2+3 94(81.7) 21(18.3) 94(81.7) 21(18.3) 97(84.4) 18(15.6) 69(60.0) 46(40.0) 52(45.2) 63(54.8)

Ki67 expression levels*^6^

Low 21(42.9) 28(57.1) <0.0001 20(40.8) 29(59.2) <0.0001 25(51.0) 24(49.0) <0.0001 37(75.5) 12(24.5) 0.0821 24(49.0) 25(51.0) 0.5074

High 110(79.1) 29(20.9) 109(78.4) 30(21.6) 117(84.2) 22(15.8) 84(60.4) 55(39.6) 60(43.2) 79(56.8)

Subtypes*^7^

TN 41(85.4) 7(14.6) 0.0011 41(85.4) 7(14.6) 0.0005 41(85.4) 7(14.6) 0.0019 19(39.6) 29(60.4) 0.0008 16(33.3) 32(66.7) 0.2611

Luminal A 15(48.4) 16(51.6) 14(45.2) 17(54.8) 16(51.6) 15(48.4) 26(83.9) 5(16.1) 17(54.8) 14(45.2)

Luminal B 36(73.5) 13(26.5) 35(71.4) 14(28.6) 39(79.6) 10(20.4) 34(69.4) 15(30.6) 25(51.0) 24(49.0)

Luminal-HER2 20(55.6) 16(44.4) 20(55.6) 16(44.4) 25(69.4) 11(30.6) 25(69.4) 11(30.6) 14(38.9) 22(61.1)

HER2 27(81.8) 6(18.2) 27(81.8) 6(18.2) 30(90.9) 3(9.1) 23(69.7) 10(30.3) 16(48.5) 17(51.5)

Chemotherapy regimen

Taxane 30(62.5) 18(37.5) 0.3691 29(60.4) 19(39.6) 0.3534 32(66.7) 16(33.3) 0.1649 34(70.8) 14(29.2) 0.5582 26(54.2) 22(45.8) 0.0912

Anthracycline and taxane 103(72.5) 39(27.5) 101(71.1) 41(28.9) 112(78.9) 30(21.1) 88(62.0) 54(38.0) 57(40.1) 85(59.9)

Others 6(66.7) 3(33.3) 7(77.8) 2(22.2) 8(88.9) 1(11.1) 6(66.7) 3(33.3) 26(54.2) 22(45.8)

*^1^ low < 3.664, high ≥ 3.664; *^2^ low < 3.279, high ≥ 3.279; *^3^ low < 1.782, high ≥ 1.782 ; *^4^ low < 4.416 , high ≥ 4.416 ; *^5^ low < 20.138 , high ≥ 20.138; *^6^ low < 20%, high ≥ 20%; *^7^ TN, estrogen receptor (ER)-negative/human epidermal growth factor receptor 2 (HER2)-negative; Luminal A, ER-positive/HER2-negative with Ki67< 20%; Luminal B, ER-positive/HER2-negative with Ki67 ≥ 20%, Luminal-HER2, ER-positive/HER2-positive; HER2, ER-negative/HER2-positive.
