# Supplementary material for: Significance of Metabolic Tumor Volume at Baseline and Reduction of Mean Standardized Uptake Value in 18F-FDG-PET/CT Imaging for Predicting Pathological Complete Response in Breast Cancers Treated with Preoperative Chemotherapy
Source: Ann Surg Oncol. 2019 Apr 2;26(7):2175–83. doi: 10.1245/s10434-019-07325-8 (PMC6545174; doi:10.1245/s10434-019-07325-8)
Supplement: Supplementary file 4 — Supplementary material 4 (DOCX 24 kb) [file 10434_2019_7325_MOESM4_ESM.docx]

Supplementary Table 3 Pathological complete response (pCR) rates according to the combination of MTV at baseline*^1^ and ∆SUVmean*^2^ in FDG*^3^ accumulation-positive and -negative breast cancers after treatment

MTV and ∆SUVmean pCR*^2^ Non-pCR

All breast cancers

MTV-low and ∆SUVmean-high 5 (100%) 0 (0%)

MTV-high and ∆SUVmean-low 1 (12.5%) 7 (87.5%)

Others 5 (55.6%) 4 (44.4%)

FDG uptake-positive after treatment

MTV-low and ∆SUVmean-high 0 (0%) 0 (0%)

MTV-high and ∆SUVmean-low 0 (0%) 5 (100%)

Others 0 (0%) 1 (100%)

FDG uptake-negative after treatment

MTV-low and ∆SUVmean-high 5 (100%) 0 (0%)

MTV-high and ∆SUVmean-low 1 (33.3%) 2 (66.7%)

Others 5 (62.5%) 3 (37.5%)

*^1^ low < 4.416, high ≥ 4.416; ^*2^ low < -55.8, high ≥ -55.8; *^3 18^F-fluorodeoxyglucose.
