# Supplementary figures and images for: Significance of Metabolic Tumor Volume at Baseline and Reduction of Mean Standardized Uptake Value in 18F-FDG-PET/CT Imaging for Predicting Pathological Complete Response in Breast Cancers Treated with Preoperative Chemotherapy
Source: Ann Surg Oncol. 2019 Apr 2;26(7):2175–83. doi: 10.1245/s10434-019-07325-8 (PMC6545174; doi:10.1245/s10434-019-07325-8)

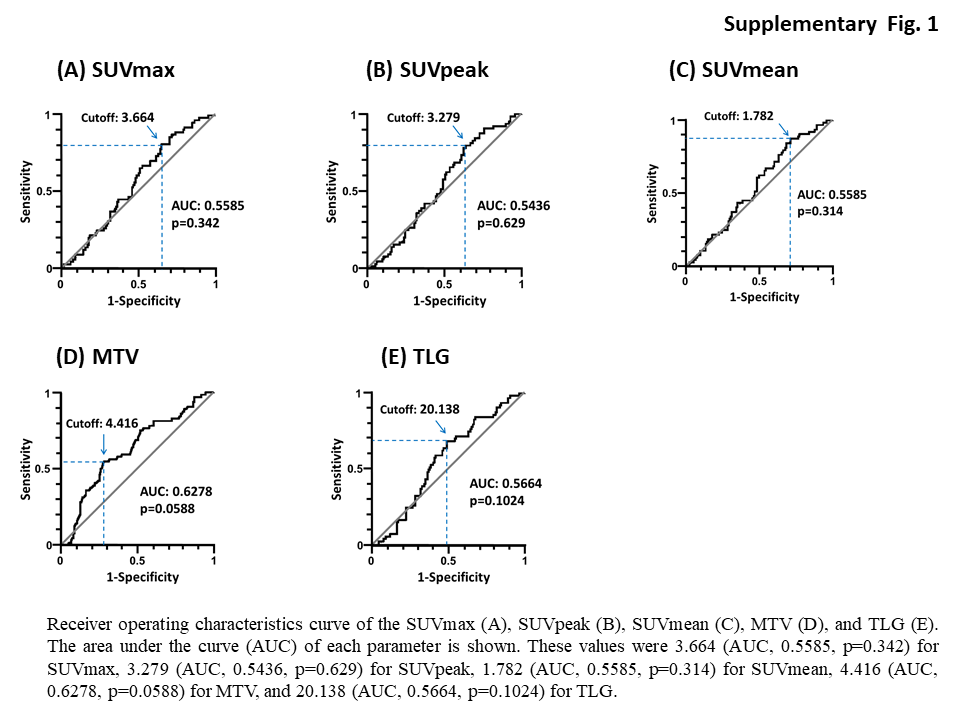

Supplement: Supplementary file 5 — Supplementary material 5 (TIFF 134 kb) [file 10434_2019_7325_MOESM5_ESM.tif]

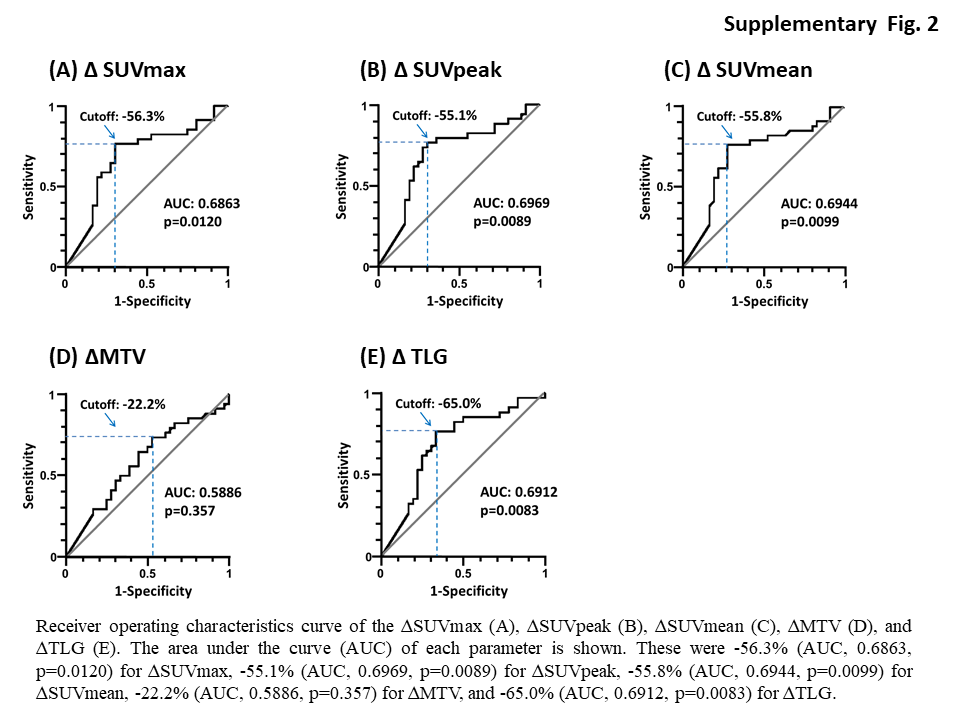

Supplement: Supplementary file 6 — Supplementary material 6 (TIFF 143 kb) [file 10434_2019_7325_MOESM6_ESM.tif]

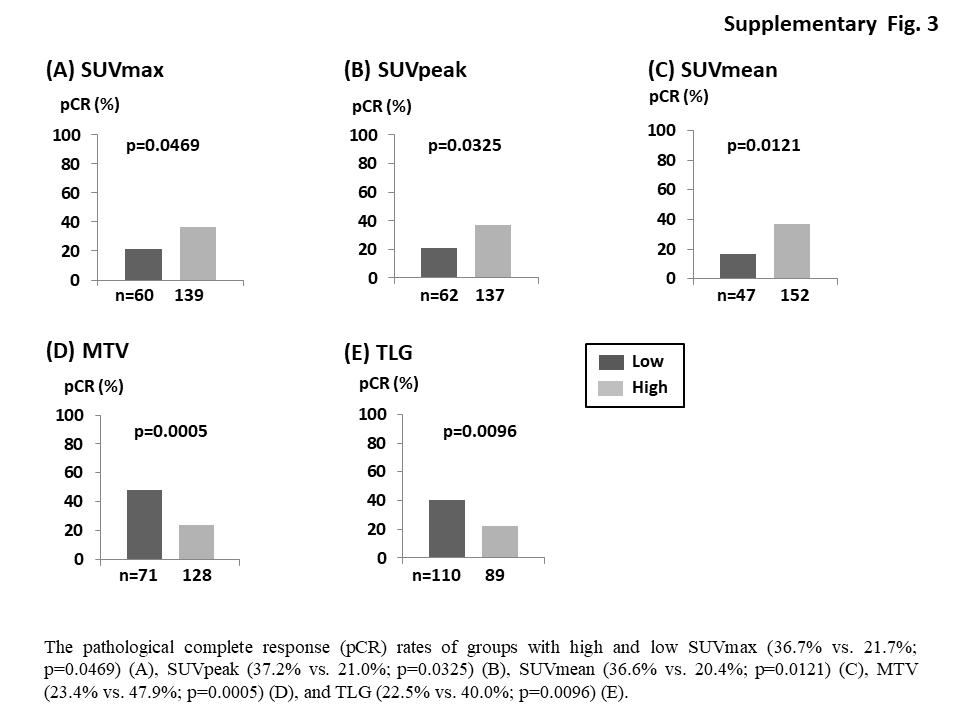

Supplement: Supplementary file 7 — Supplementary material 7 (TIFF 67 kb) [file 10434_2019_7325_MOESM7_ESM.tif]

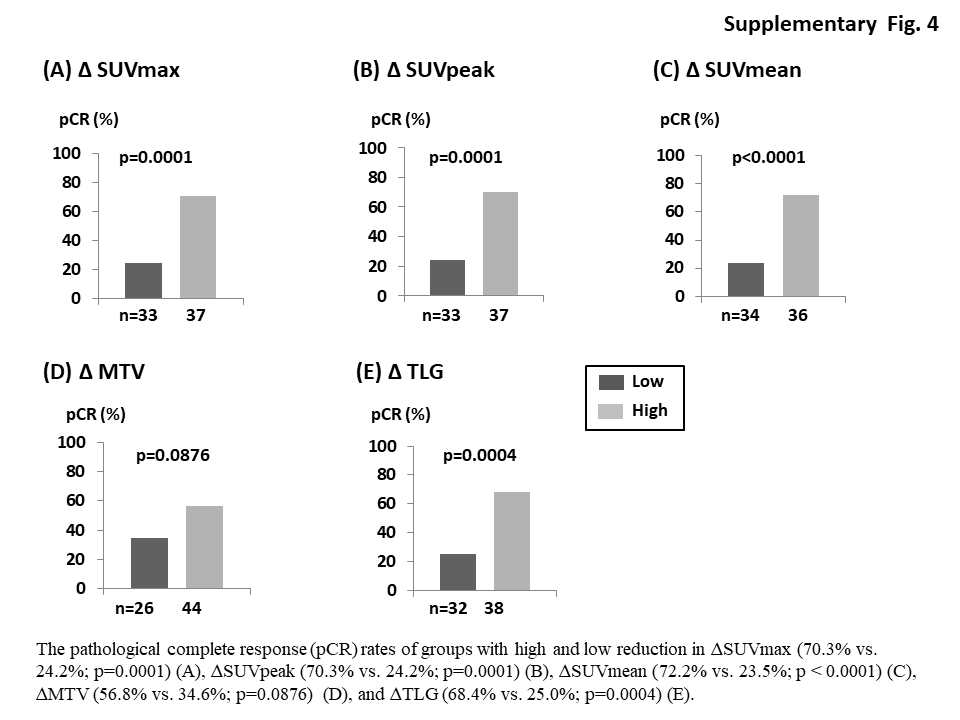

Supplement: Supplementary file 8 — Supplementary material 8 (TIFF 70 kb) [file 10434_2019_7325_MOESM8_ESM.tif]
